# Dataset of infected date palm leaves for palm tree disease detection and classification

**DOI:** 10.1016/j.dib.2024.110933

**Published:** 2024-09-11

**Authors:** Abdallah Namoun, Ahmad B. Alkhodre, Adnan Ahmad Abi Sen, Yazed Alsaawy, Hani Almoamari

**Affiliations:** a AI Centre, Faculty of Computer and Information Systems, Islamic University of Madinah, Madinah 42351, Saudi Arabia; b Smart Cities, University of Prince Mugrin, Al-Madinah, Saudi Arabia

**Keywords:** Date palm tree, Phoenix dactylifera, Palm leaf diseases, Image dataset, Plant leaf classification, Deep learning, CNN

## Abstract

This article presents an image dataset of palm leaf diseases to aid the early identification and classification of date palm infections. The dataset contains images of 8 main types of disorders affecting date palm leaves, three of which are physiological, four are fungal, and one is caused by pests. Specifically, the collected samples exhibit symptoms and signs of potassium deficiency, manganese deficiency, magnesium deficiency, black scorch, leaf spots, fusarium wilt, rachis blight, and parlatoria blanchardi. Moreover, the dataset includes a baseline of healthy palm leaves. In total, 608 raw images were captured over a period of three months, coinciding with the autumn and spring seasons, from 10 real date farms in the Madinah region of Saudi Arabia. The images were captured using smartphones and an SLR camera, focusing mainly on inflected leaves and leaflets. Date palm fruits, trunks, and roots are beyond the focus of this dataset. The infected leaf images were filtered, cropped, augmented, and categorized into their disease classes. The resulting processed dataset comprises 3089 images. Our proposed dataset can be used to train classification deep learning models of infected date palm leaves, thus enabling the early prevention of palm tree-related diseases.

Specifications Table

**Table:** 

Subject	Computer Science, Artificial Intelligence, Agricultural Sciences, Agronomy and Crop Science, Plant Science
Specific subject area	Computer Vision, Pattern Recognition, Object Detection, Image Classification, Deep Learning, Automated Harvesting
Type of data	Images (.jpg, .jfif, .heic) Raw, Filtered, Analyzed.
Data collection	Images of infected palm leaves and leaflets were captured in natural environments using six smartphones and a digital camera with the following technical specifications: • iPhone 15 Pro: 48MP Main: 24 mm, ƒ/1.78 aperture, sensor‑shift optical image stabilization, 100 % focus pixels. • iPhone 14: 12MP Main: 26 mm, ƒ/1.5 aperture, sensor‑shift optical image stabilization, 100 % focus pixels. • iPhone 13 Pro Max: 12 MP, f/1.5, 26 mm (wide), 1/1.7″, 1.9 µm, dual pixel PDAF, sensor-shift OIS. • iPhone 13 Pro: 12 MP, f/1.5, 26 mm (wide), 1/1.7″, 1.9 µm, dual pixel PDAF, sensor-shift OIS. • iPhone 13: 12 MP, f/1.6, 26 mm (wide), 1/1.9″, 1.7 µm, dual pixel PDAF, sensor-shift OIS. • iPhone 12 Pro Max: 12 MP, f/1.6, 26 mm (wide), 1/1.9″, 1.7 µm, dual pixel PDAF, sensor-shift OIS. • Nikon D5300: 24.2 MP, Sensor size 366.6mm2 (23.50 mm x 15.60 mm), 3.92 µm, ISO Sensitivity ISO 100 −12,800. The images of palm leaves were collected from 10 real date farms in Madinah during the period spanning November to December 2023 and May 2024. The images were shot in daylight (from 9am to 5pm) with natural environment conditions.
Data source location	City: Medina or Al-Madinah al-Munawwarah, Province: Medina Province, Country: Saudi Arabia. Date fruit farms are located within this range of coordinates: 24° 28′ 12″ N, 39° 36′ 36″ EThe specific details of the farms’ locations are defined as follows: • Date Farm 1 (39.6161549 24.5039035) • Date Farm 2 (39.6388775 24.4534438) • Date Farm 3 (39.3964722 24.6075756) • Date Farm 4 (39.5975378 24.3335346) • Date Farm 5 (39.5707086 24.4741009) • Date Farm 6 (39.6284975 24.4419179) • Date Farm 7 (39.6844226 24.4258807) • Date Farm 8 (39.6728144 24.4303728) • Date Farm 9 (39.5709808 24.4760874) • Date Farm 10 (39.6177167 24.4538728) Figure 1 depicts the map of Madinah and the locations of the date palm farms from which images were taken.
Data accessibility	Repository name: Mendeley Data Data identification number: 10.17632/g684ghfxvg.2 Direct URL to data: https://data.mendeley.com/datasets/g684ghfxvg/2

## 1 Value of the Data

• The proposed dataset helps to overcome the lack of images characterizing various categories of palm leaf diseases. In fact, there is a limited number of public data on infected palm leaves to support both research and industry works. In the background section, we highlight a few related works and clarify our fundamental differences.
• The image dataset adds to existing date palm tree datasets to support research works in the fields of agriculture, plant pathologies, and bioinformatics. Our dataset showcases four types of leave infections, enabling a better understanding of the dynamics of these infections in date farms. Moreover, leaf infections are exemplified in the image data by different signs, symptoms, and patterns of abnormalities (e.g., color of leaves, severity, etc.).
• The dataset will be used by researchers in the fields of machine learning (ML) and deep learning (DL) to develop, test, and improve classification models of date palm pathologies and infections affecting leaves. Deep learning models trained on our dataset and similar datasets will form the basis for automated palm leaf disease diagnosis systems. Unlike manual methods of palm tree disease detection, DL models are expected to save human and financial resources efficiently during date cultivation.
• The dataset can be used by other researchers for comparison with other public palm tree datasets to identify patterns of infections and genre of diseases and its possible relationship with various palm species and regions.
• The dataset may be used to complement the development of automatic palm leaf disease detection and classification systems and thereby contribute toward the implementation of smart date farms. Early identification of leaf infections will empower farm owners and policymakers to devise more effective plant disease control and management and palm cultivation.

## 2 Background

Saudi Arabia has more than 31 million palm trees, contributing approximately 17 % of the world's production of date fruit [1]. The Madinah region, located in the west of Saudi Arabia, has more than 26,000 date farms, generating more than 253 million US dollars in the year 2023 [2]. However, palm tree parts (e.g., root, trunk, leaves, and fruits) can be infected by various contagious diseases originating from fungal and bacterial pathogens. Detecting palm tree diseases at an early stage is crucial to overcome the disease and this may be achieved using various of variants of deep learning algorithms, such as Artificial Neural Network (ANN), Convolutional Neural Network (CNN) and Recurrent Neural Network (RNN) [3]. Typically, these models ought to be trained on large datasets to produce optimal plant disease predictions. However, datasets of date palm trees are scarce.

Recent publicly available datasets focused mainly on images of date fruits. Authors in [4] presented a date fruit dataset collected from Riyadh, Saudi Arabia. 8079 images featuring five types of date (i.e., Naboot Saif, Khalas, Barhi, Meneifi, and Sullaj) were shot in bunches or individually. The images represented all date maturity stages. Similarly, authors in [5] collected images and videos of four main types of dates (i.e., Majhoul, Boufaguos, Kholt, and Bouisthami) under diverse daylight conditions to support date harvesting operations. The dataset was collected from farms in Morocco and includes 9096 dates in four maturity stages (i.e., Immature, Khalal, Rutab, and Tamar stage). Authors in [6] presented a dataset of 3004 images of diverse types (i.e., Aseel, Fasli Toto, Gajar, and Kupro dates), sizes (i.e., large, medium, and small) and grades (grade 1, grade 2, and grade 3) of dates, collected from a village in Pakistan. This dataset aims to train deep learning models for the automatic classification of date fruit varieties.

However, datasets featuring diseases and infections affecting date palm trees are quite limited. One recent dataset is published by [7], which includes 3000 palm leaf images representing four categories of dubas insect infections (i.e., healthy, bug, honey, and mixed). This leaf dataset was collected from Iraq and is restricted to infestations caused by the dubas insect. Our dataset complements the dubas dataset by covering a more comprehensive range of palm leaf pathologies caused by other factors (e.g., nutrient deficiencies, fungus). Moreover, our dataset is the second study to cover the region of Madinah, Saudi Arabia. Authors in [8] published a dataset covering one palm disease, namely the phytoplasma disease (i.e., Wijam), which causes a lethal yellowing of palm trees in the Madinah region. Typically, diverse geographical areas might affect the symptoms and severity of date palm disorders differently. As such, our unique image dataset extends the dubas dataset by covering eight leaf pathologies and enables cross-country comparison (e.g., Saudi Arabia Vs. Iraq) and early date palm disease detection and management.

## 3 Data Description

Our article describes the date palm leaves dataset, featuring eight types of physiological, fungal and insect induced diseases, which were captured from 10 different date fruit farms in Madinah. The final dataset contains a total of 3697 images and is organized within two main folders. The first folder contains nine subfolders of raw images, and the second folder contains nine subfolders of augmented/processed images. The raw images folder contains 608 images, occupying a total of 891 MB. However, the analyzed images folder contains 3089 images occupying a total of 198 MB. The specifics of the dataset and the palm leaf pathologies are listed in Table 1.

**Table 1:** The number of date palm leaves per disease type (as colored images) in our dataset.

	Disease Type								Healthy	Total
	Physiological			Fungal				Insect		
	K-	Mn-	Mg-	Black Scorch	Leaf Spots	Wilt Fusarium	Rachis Blight	Parlatoria blanchardi		
Raw / Original	89	16	55	82	68	102	100	60	36	608
Analyzed	831	415	566	106	69	210	402	101	389	3089
Total	920	431	621	188	137	312	502	161	425	3697

The images were shot using six smartphones and one SLR camera, with the resolution of raw images ranging from 2778×1284 to 6000×4000 pixels. However, the resolution of processed images is set to 300×300 pixels. All leaf images in our dataset were shot at distances ranging between 15 and 100CM. Moreover, the infected palm leaf images were captured from different angles and positions under diverse illumination conditions. Such a variety of palm leaf infections enables comprehensive training and testing of smart palm disease detection and management systems.

**Fig. 1:**
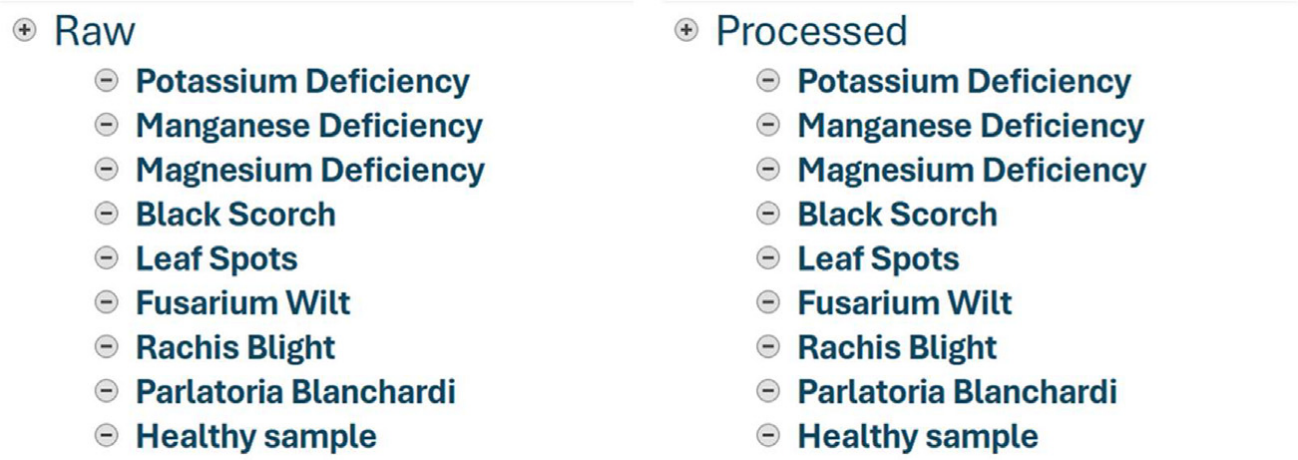
The folder structure of our infected date palm leaves dataset.

The content of our dataset is deposited into the Mendeley Data repository [9] with the following simple folder structure:

The above sub-folders (in Fig 1) represent the subsequent palm leaf disorders and pathologies. A sample of each disease category is depicted in Table 2.

• **Disease one (Potassium Deficiency)**: this folder contains a total of 831 augmented leaf images. The disease presented in this sample is a physiological disorder resulting from the lack of potassium (K) in soil. It usually starts appearing on the older leaves and leaflets. The leaf symptoms exhibited by potassium deficiency are very similar to those of manganese deficiency and can be easily confused. However, in this type of disorder, discoloration (e.g., translucent yellow-orange) is confined to the leaflet tips, while the green area of the leaves is large [10]. Such a type of disease is not infectious as it is not caused by a specific pathogen.
• **Disease two (Manganese Deficiency)**: this folder contains a total of 415 augmented leaf images. The disease presented in this sample is a physiological disorder resulting from the lack of Manganese (Mn) or a high pH level in soil. Initially, manganese deficiency causes paleness/yellowing starting at the base of older leaves, followed by dryness and death of palm tissues [10]. Symptoms include the prevalence of extended dead areas or streaks along the veining of the leaf and then the withering of leaflets.
• **Disease three (Magnesium Deficiency)**: this folder contains a total of 566 augmented leaf images. The disease presented in this sample is a physiological disorder resulting from the lack of magnesium (Mg) in the soil. Magnesium-deficient leaves exhibit wide yellow bands along the edges of the older leaves and fronds [10]. Generally, the tip of leaves dries and dies first, while the central portions remain green.
• **Disease four (Black Scorch)**: this folder contains a total of 106 augmented leaf images. The disease presented in this sample shows the occurrence of rough brown to black spots and burns on the palm leaves, rachis, and its structure. This disease is commonly referred to as black scorch (also known as Medjnoon or fool's disease), and its morphological symptoms may include the deformation and twisting of infected leaves, among others. This disease is caused by fungal infections, including Thielaviopsis paradoxa and Chalaropsis radicicola [10]. If left untreated, the black scorch eventually leads to the death of date palm trees after a few years of invasion.
• **Disease five (Leaf Spots)**: this folder contains a total of 69 augmented leaf images. The disease presented in this sample is known as the leaf spots disease, a common disorder that occurs particularly in date palm farms exposed to low temperatures for a long time or humid weather. Various fungal species (e.g., Bipolaris, Cladosporium herbarum, Alternaria alternata) may attack palm leaves of all ages, causing variable leaf spots symptoms (e.g., brown, reddish-brown, or brown-gray spots) [10]. Insect infestations also contribute to the spread of leaf spots disease.
• **Disease six (Fusarium Wilt)**: this folder contains a total of 210 augmented leaf images. The disease presented in this sample is called Fusarium wilt or sudden decline disease, which characterizes the sudden drying of apical leaves and rachis resembling the appearance of wet feathers. This infestation is caused by a notorious fungus called Fusarium oxysporum [10], leading to a quick death of the palm trees within months of infection.
• **Disease seven (Rachis Blight)**: this folder contains a total of 402 augmented leaf images. The disease presented in this sample is called Petiole/Rachis blight, caused by a fungal pathogen named Serenomyces califronica. This fungal infection starts by developing brown or reddish-brown lesions on the petiole of the oldest leaves and generally on only one side of the rachis before invading the second side [10].
• **Disease eight (Parlatoria Blanchardi)**: this folder contains a total of 101 augmented leaf images. The disease demonstrated in this sample is called Parlatoria blanchardi scale, caused by an abundant pest infestation [11]. Date palm scale symptoms include white or gray discoloration of the leaves starting at the base of older fronds. Parlatoria blanchardi eventually infests date fruits, causing several deformations.
• **Healthy sample**: this folder contains a total of 389 augmented leaf images. This is the only sample that comprises images of healthy date palm leaves. Typically, healthy date palms have green leaves with no evident discoloration or deformation to their fronds, as opposed to diseased palms. No physiological or pathogenic symptoms are detected in the healthy image sample.

**Table 2:** A sample of date palm leaf diseases/disorders and a healthy set.

Leaf Disorder	Example 1	Example 2
K- Deficient		
Mn- Deficient		
Mg- Deficient		
Black Scorch		
Leaf Spots		
Wilt Fusarium		
Rachis Blight		
Parlatoria blanchardi		
Healthy		

## 4 Experimental Design, Materials and Methods

**Fig. 2:**
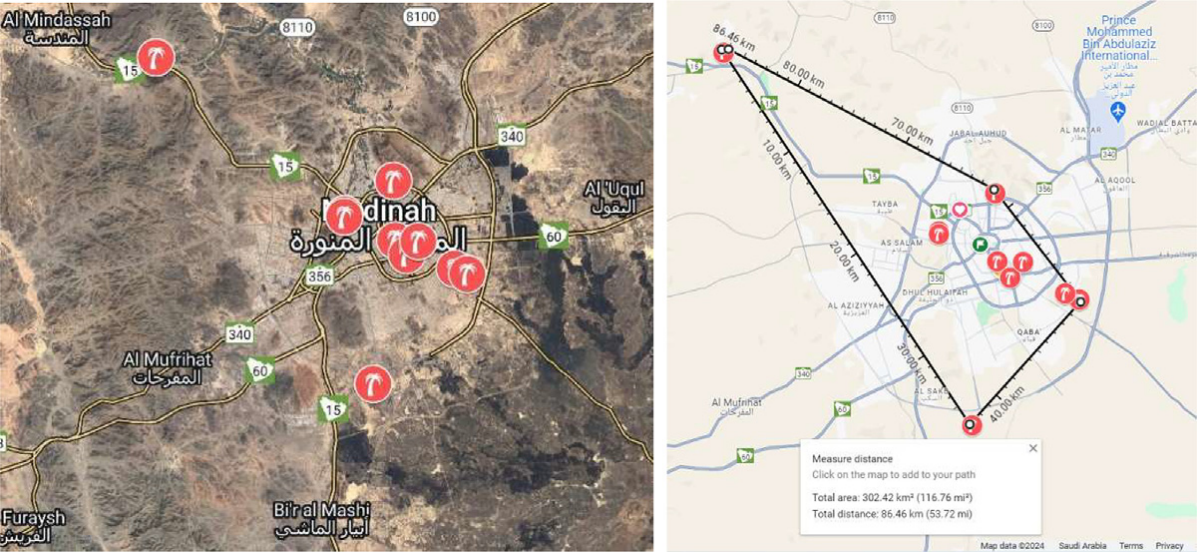
The location of date fruit farms (represented using the palm tree icon) in Madinah (Saudi Arabia) of our dataset.

The infected palm leaves dataset was collected over a period of three months (namely in the autumn and spring seasons in Madinah) from 10 date farms located in the Madinah region, Saudi Arabia. When we measured the distance using Google Maps, we found that eight farms are located approximately 14 km apart, while the remaining two farms are about 37 km apart. We selected date farms based on our acquittances and connections with the owners for convenience and availability. Therefore, it was not feasible for this study to apply a random sampling strategy during the selection of date farms. The geographic locations of the farms and their distances are specified on Google My Maps, as depicted in Fig. 2.

Smartphones and an SLR camera were used to specifically shoot diverse leave disorders in palm trees under real conditions, resulting in a total of 608 clean raw images. The raw images were classified into eight leaf diseases and a healthy sample. The images were shot from varying distances and angles. Statistics of the final set of diseases are detailed in Table 1.

**Fig. 3:**
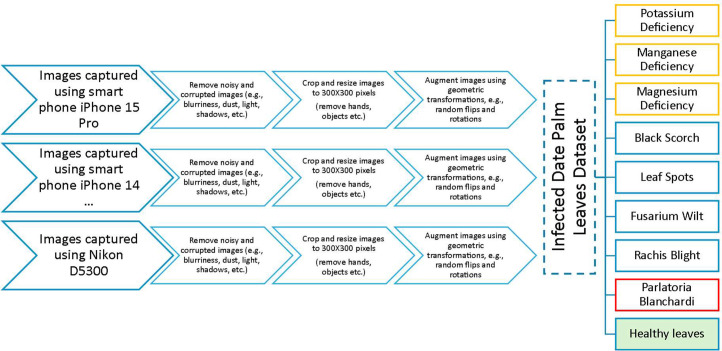
Data collection and analysis process of our infected date palm leaves dataset.

In our data collection process, depicted in Fig. 3, we started by reviewing all captured images and eliminating noisy or corrupted images (e.g., bright, dark, or blurry), which were impacted by light conditions or movement during the shooting phase. Next, we cropped the clear images to eliminate any irrelevant objects (e.g., body parts of photographers, farm and palm tree parts) and focus on the specific leaf pathologies using an image editing software (i.e., imageresizer). Our next objective was to augment the image dataset using image manipulation functions to enable efficient and cost-effective training of various deep learning models. Subsequently, we resized the cropped images to 300×300 pixels to meet the input size requirement of CNN models [12]. We augmented the processed images using various geometric operations, including rotation and flipping. The augmented dataset contains a total of 3089 images. All our images are stored in RGB format.

During the picture shooting of palm leaf diseases, we tried to photograph the infected fronds from different angles and distances to enrich our dataset as much as possible. Next, the original images were processed by following the subsequent steps:

1. We manually determined the location of the disease on each image,
2. When the image contained only one infected place, the image was cropped to the site of the disease,
3. However, if there are more than one infected location, each location is processed separately,
4. The crop has a fixed size of 300×300 pixels,
5. The resulting images are then subjected to geometric transformations, such as rotation to more than one angle.

In the original dataset, some leaf images were captured from multiple angles. We have revised all images of the original dataset and renumbered them. Therefore, images belonging to the same palm leaf are assigned the same first digit (e.g., 2.1, 2.2, 2.3). Images with different first digit belong to different fronds.

## Limitations

We acknowledge some limitations impacting the quality of our image dataset. Notably, the infected leaf sample size is small in size and collected from a handful of date farms in Madinah. Therefore, by no means does the dataset represent all date palm farms of the Madinah region nor of Saudi Arabia. The date farms were selected based on accessibility and convenience, which may have introduced a selection bias influencing the quality of palm leaves and diversity of leaf diseases. The infections depicted in our dataset are limited to eight categories only and a particular season. We therefore acknowledge that there are other palm leaf disorders, which were not explored in this work. Moreover, the lack of information regarding date farms (e.g., soil properties, date tree species, pest management practices) may influence our understanding of date palm leaf diseases and disorders. Deep learning models trained solely on our dataset may not perform well when tested on infected palm leaves taken from other regions or during other seasons (e.g., winter or summer). Finally, infected leaf symptoms range from mild to severe, and sometimes it may be challenging to identify the specific type of infection by human evaluation only. To confirm the genre of infection, one would need to perform a DNA analysis, which is beyond the scope of this project.

## Ethics Statement

The authors have read and followed the ethical requirements for publication in Data in Brief and confirm that the current work does not involve human subjects, animal experiments, or any data collected from social media platforms.

## CRediT authorship contribution statement

**Abdallah Namoun:** Conceptualization, Investigation, Methodology, Writing – original draft, Writing – review & editing. **Ahmad B. Alkhodre:** Conceptualization, Methodology, Writing – review & editing, Supervision, Funding acquisition. **Adnan Ahmad Abi Sen:** Conceptualization, Investigation. **Yazed Alsaawy:** Conceptualization, Investigation, Writing – review & editing. **Hani Almoamari:** .

## Data Availability

Diseases of date palm leaves dataset (Original data) (Mendeley Data).
